# Working in the woodlands: a mixed methods evaluation of Green Care in first episode psychosis

**DOI:** 10.1192/bjo.2021.184

**Published:** 2021-06-18

**Authors:** Harriet Sharp, Sharon Cuthbert, Clio Berry

**Affiliations:** 1 Sussex Partnership Nhs Foundation Trust, Brighton and Sussex Medical School; 2 Sussex Partnership Nhs Foundation Trust; 3 Brighton and Sussex Medical School University of Sussex

## Abstract

**Aims:**

Recognition of the essential role of nature-based activities for general wellbeing is expanding. Previous evaluation of nature-based activities has shown that those with greater mental health needs may benefit proportionally more compared to the general population. Currently, there is limited evidence of the benefits of green care for those with severe and enduring mental illness, including psychosis.

We aim to establish benefits and difficulties encountered during a 10-session green care programme for 18-30 year olds who have experienced first episode of psychosis (FEP) using a mixed methods approach.

**Method:**

This was a service evaluation of the Woodland Group, run by Circle of Life Rediscovery (CLR) and commissioned by Sussex Partnership NHS Foundation Trust in Autumn 2019 for 10 half-day sessions. All participants were aged 18–30 years, referred from Early Intervention in Psychosis service and had experienced FEP. Patients were supported by EIS staff with a ratio of at least 3:1. Sessions consisted of a welcome and agenda setting, ice-breaking activity, core nature-based activity (such as roasting chestnuts, maintaining the woodland area) and a ‘sense meditation’.

Quantitative data for this evaluation were collected through routinely collected 15-item Questionnaire on the Process of Recovery (QPR), and a semi-structured intervention experience questionnaire. Qualitative data were collected via a focus group within the final session of the Woodlands Group. Thematic analysis was performed by the three co-authors.

**Result:**

Session attendance ranged between 3-15. 4/8 patients showed reliable improvement on QPR outcome measures, 1 showed deterioration and 3 showed no change. Mean QPR scores showed modest increase from average 3.4 (week 1) to 3.8 (week 10). 100% of respondents would recommend this group to others. Thematic analysis identified themes of connection with nature and others, development of a sense of wellbeing and ‘peacefulness’ and new perspectives on psychotic experience.

**Conclusion:**

This small, retrospective evaluation is the first to investigate green care interventions for young people experiencing FEP. Our results reflect the positive informal feedback from participants and supporting staff following attendance at the Woodlands Group. Limitations include small sample size, incomplete data, and reliance on patient-reported outcomes. These findings show promise for green care activities within EIS and represents a sustainable intervention in mental health care.

